# RNA processing: a new player of genomic instability in multiple myeloma

**DOI:** 10.18632/oncoscience.361

**Published:** 2017-09-21

**Authors:** Matteo Marchesini, Elena Fiorini, Simona Colla

**Affiliations:** Department of Leukemia, The University of Texas MD Anderson Cancer Center, Houston, TX 77030, USA

**Keywords:** genomic instability, RNA-binding proteins, multiple myeloma, DNA damage

Genomic instability, a hallmark of almost all cancers, originates from the combined effects of a deregulated DNA damage response, DNA repair defects, and a failure of cell-cycle checkpoints before the damaged DNA is propagated to daughter cells [1]. Alterations in this network of genomic integrity–preserving pathways lead to the accumulation of mutations, aneuploidy, and chromosomal alterations, the main causes of cancer. Multiple myeloma (MM) is a clonal malignancy of terminally differentiated plasma cells that reside and expand in the bone marrow. MM cells are characterized by a high aneuploidy incidence and recurrent structural chromosomal alterations, features that reflect these cells’ underlying genomic instability [2].

Recent evidence shows that elevated homologous recombination (HR) activity is a key mediator of genomic instability in MM. Increased HR activity results in an increased burden of mutations and the continued accumulation of genetic alterations, thereby leading to drug-resistant MM phenotypes and disease progression [3]. Although activated HR plays a critical role in the clonal evolution of MM by contributing to survival mechanisms, it also creates therapeutically exploitable vulnerabilities by inducing oncogene addiction [4] and selective sensitivity to specific anti-MM agents. An example is represented by the inhibition of the 26S proteasome that impairs HR-mediated repair of DNA Damage in MM cells and results in a contextual synthetic lethality when combined with PARP inhibitors [5].

Post-transcriptional RNA processing adds another layer of complexity to the maintenance of genomic stability in MM. RNA processing includes the concerted modification of the splicing patterns of transcripts involved in the repair of DNA damage and those involved in the regulation of genomic stability, which occurs in reponse to genotoxic stresses [6]. The alternative splicing reprogramming that follows the DNA damage response relies on the regulation of the expression, localization, and activity of RNA-binding proteins (RBPs), which directly bind specific pre-mRNA and mRNA sequences and act as gatekeepers of genomic integrity [7]. The disruption of the regulatory interplay between RBPs and the DNA damage response inexorably promotes genomic instability and tumorigenesis, thereby contributing to drug-resistant phenotypes. Therefore, the targeting of aberrant RBP functions during the DNA damage response is an active area of preclinical investigation that may lead to the development of therapeutic approaches to sensitize MM and other cancer cells to DNA-damaging agents.

The potential to therapeutically target aberrant RBP activities has been illustrated by our recent studies, which show that highly genomically unstable and aggressive MMs carrying the 1q21 amplification have acquired dependency on the 1q21 amplification–induced overexpression of the RBP interleukin enhancer binding factor 2 (ILF2) [8]. ILF2 is a key modulator of the HR DNA repair pathway, which enables the tolerance of genomic instability, enhances cell survival, and promotes adaptive mechanisms to genotoxic stress in a dose- dependent manner, explaining why 1q21 patients benefit less from high-dose therapy than non–1q21 patients do. At the mechanistic level, we have demonstrated that high ILF2 expression levels drive resistance to genotoxic agents by modulating YB1's cytoplasmic-to-nuclear translocation and its interaction with the splicing factor U2AF65, which promotes the mRNA splicing of transcripts involved in HR in response to DNA damage. This observation is consistent with our clinical studies showing that nuclear expression of ILF2 is strongly correlated with that of YB1 in 1q21 MMs and that YB1 downregulation during DNA damage activation increases γH2AX accumulation and caspase 3 activation to an extent similar to that of ILF2 depletion. Our findings suggest that ILF2 has clinical utility as a biomarker of more aggressive MM and support the development of strategies for blocking the ILF2/YB1/HR signaling axis to enhance the effectiveness of current therapies based on DNA-damaging agents.

**Figure 1:**
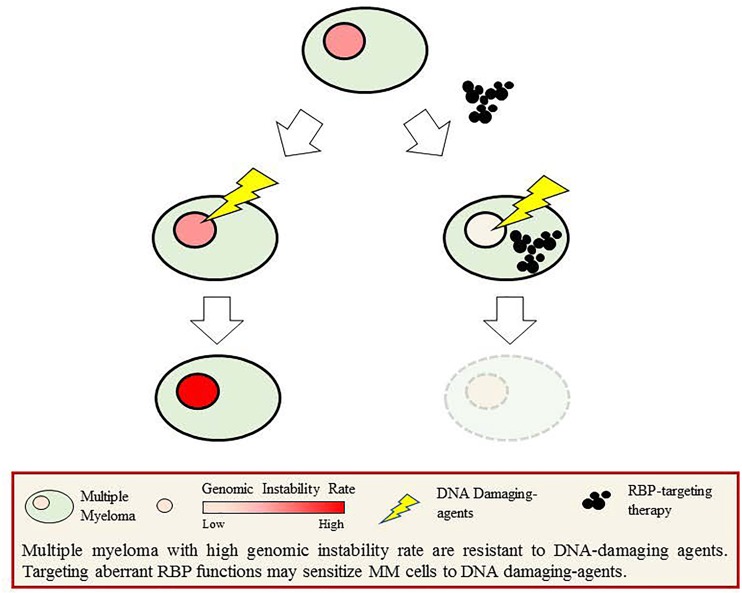
Multiple myeloma with high genomic instability rate are resistant to DNA-damaging agents

Further dissecting the altered function of RBPs and RBP-associated RNAs in response to DNA damage will provide a deeper insight into the mechanisms driving genomic instability in MM and lead to the identification of selective vulnerabilities that can be used to target highly genomically unstable cancers (Figure 1).
